# Innovations in the Assessment of Primary and Secondary Raynaud’s Phenomenon

**DOI:** 10.3389/fphar.2019.00360

**Published:** 2019-04-16

**Authors:** Barbara Ruaro, Vanessa Smith, Alberto Sulli, Carmen Pizzorni, Samuele Tardito, Massimo Patané, Sabrina Paolino, Maurizio Cutolo

**Affiliations:** ^1^Research Laboratory and Academic Division of Clinical Rheumatology, Department of Internal Medicine (Di.M.I.), San Martino Polyclinic Hospital, University of Genova, Genova, Italy; ^2^Department of Rheumatology, Ghent University Hospital, Ghent, Belgium; ^3^Department of Internal Medicine, Ghent University, Ghent, Belgium; ^4^Unit for Molecular Immunology and Inflammation, VIB Inflammation Research Center (IRC), Ghent, Belgium

**Keywords:** Raynaud’s phenomenon, systemic sclerosis, microvascular damage, nailfold videocapillaroscopy, peripheral blood perfusion, laser techniques

## Abstract

**Objectives:** Raynaud’s phenomenon (RP) is characterized by intense vasospasm of the digital arteries that causes characteristic color changes in fingers. There are two main types of RP: Primary RP (PRP) and Secondary RP (SRP). PRP is a benign condition. Whilst SRP is associated with several connective tissue diseases (CTD), in particular systemic sclerosis (SSc). The objectives of this report were: to present a short review on morphological (nailfold videocapillaroscopy, NVC) and functional techniques (laser tools and thermography) that allow for a correct diagnosis and treatment of RP and to investigate blood perfusion (BP) by laser speckle contrast analysis (LASCA) in different skin areas of hands and face in PRP, SRP to SSc, and healthy subjects (CNT).

**Methods:** 31 PRP patients (LeRoy criteria), 70 SRP to SSc (ACR/EULAR criteria) and 68 CNT were enrolled. BP was assessed by LASCA at the level different areas of hands and face. NVC was performed to distinguish between PRP and SRP, and to detect the proper pattern of nailfold microangiopathy in SSc patients.

**Results:** Both PRP and SRP showed a statistically significant lower BP than CNT at the level of fingertips (*p* < 0.0001), periungual (*p* < 0.0001), palmar aspect of 3rd finger (*p* < 0.0001), and palm areas (*p* < 0.0001). Moreover, BP was significantly lower in PRP than in SRP to SSc with the “Early” pattern of microangiopathy in the same areas as above (*p* < 0.04).

**Conclusion:** By considering a small cohort of patients, BP of hands was found lower in PRP than in SSc patients with the “Early” NVC pattern of microangiopathy.

## Short Review and Introduction

Raynaud’s phenomenon (RP), first described in 1862 by Maurice Raynaud (Fardoun et al., 2016; Wigley and Flavahan, 2016), is present in 5–10% of the world’s population. It is a clinical consequence of recurrent vasospasm of the small arteries and arterioles of the fingers and toes triggered by cold or even emotional stress (Wigley and Flavahan, 2016), at times also affecting the nose, ears, or lips (Block and Sequeira, 2001; Herrick, 2005; McMahan and Wigley, 2010; Hughes and Herrick, 2016). The skin usually turns white (ischemia), blue (deoxygenation) and then red (reperfusion) (Block and Sequeira, 2001; Herrick, 2005; McMahan and Wigley, 2010; Hughes and Herrick, 2016).

There are two main categories, i.e., Primary (PRP) and Secondary RP (SRP) and most are PRP that have an isolated finding if there is no underlying pathology (idiopathic). SRP is present in various conditions, like connective tissue diseases (CTD), such as systemic sclerosis (SSc).

Ninety percent of SSc patients have RP which is the most common presenting feature and may precede diagnosis by many years (Block and Sequeira, 2001; Herrick, 2005; McMahan and Wigley, 2010; Hughes and Herrick, 2016). The suggested criteria for PRP include symmetric attacks, the absence of tissue necrosis, ulceration or gangrene, the absence of a secondary cause, negative tests for antinuclear antibodies and a normal erythrocyte sedimentation rate (LeRoy and Medsger, 1992, 2001).

When PRP diagnosis is made no underlying disease has yet been identified, making prediction of if and when it may turn into SRP difficult (Ingegnoli et al., 2010; Avouac et al., 2011; Bernero et al., 2013; Cutolo et al., 2017a). Nailfold video-capillaroscopy (NVC) is able to distinguish SRP from both PRP and healthy subjects by detecting morphological microcirculation abnormalities (Block and Sequeira, 2001; Cutolo et al., 2003; Herrick, 2005; Ingegnoli et al., 2017; Pizzorni et al., 2017b; Herrick and Murray, 2018). Follow-up nailfold capillaroscopic analysis should be performed every 6 months in PRP patients (Cutolo et al., 2003; Bernero et al., 2013).

Nailfold capillaries in PRP are usually normal in shape without any specific alterations (Ingegnoli et al., 2013; Smith et al., 2016a) or abnormal capillaroscopic findings, i.e., giant capillaries and microhemorrhages, whilst their presence is diagnostic for the “Early” NVC pattern of scleroderma microangiography (Cutolo et al., 2003, 2017a; Bernero et al., 2013; Ingegnoli et al., 2013; Smith et al., 2016a).

Indeed, abnormal nailfold capillaroscopic images (more specifically “scleroderma patterns”) were included in the 2013 European League Against Rheumatism and American College of Rheumatology classification criteria for SSc to this aim (van den Hoogen et al., 2013).

Digital vasculopathy is structural and functional in SRP due to SSc. NVC cannot measure blood perfusion (BP) under standard conditions (Mugii et al., 2009) but other techniques, like laser and thermography as well as emerging technologies are able to evaluate and quantify skin blood flow and perfusion in SSc (Wigley et al., 1990; Clark et al., 2003; Murray et al., 2009; Rosato et al., 2009, 2011; Cutolo et al., 2010, 2014, 2018b; Pauling et al., 2012a,b, 2015; Della Rossa et al., 2013; Ruaro et al., 2014, 2016, 2017b, 2018c; Sulli et al., 2014; Lambrecht et al., 2016; Wilkinson et al., 2018). Laser Doppler flowmetry (LDF) evaluates blood flow at a single skin point, providing an index of skin perfusion (Cutolo et al., 2010, 2014; Ruaro et al., 2017b, 2018c).

Laser Doppler imaging (LDI) may also be used to evaluate the microcirculatory blood flow (Wigley et al., 1990; Clark et al., 2003; Murray et al., 2009; Rosato et al., 2009, 2011). LDI assesses more than one area and is more effective than a single probe Doppler (Wigley et al., 1990; Clark et al., 2003; Murray et al., 2009; Rosato et al., 2009, 2011). LDI can help to differentiate between PRP and patients with SRP to scleroderma (Wigley et al., 1990; Clark et al., 2003; Murray et al., 2009; Rosato et al., 2009, 2011). Although Murray et al. suggested that combining laser Doppler with other imaging modalities (e.g., nailfold capillaroscopy and thermal imaging) is more effective than laser Doppler alone, these functional imaging tools are not yet widely available (Murray et al., 2009).

Laser speckle contrast analysis (LASCA) can quantify the blood flow over a defined area and is based on the principle that when laser light illuminates a tissue it forms a speckle pattern (Della Rossa et al., 2013; Ruaro et al., 2014; Lambrecht et al., 2016; Cutolo et al., 2018b). Changes in this pattern are analyzed by software and the static areas show a stationary speckle pattern, in contrast with the moving objects like red blood cells that cause the speckle pattern to fluctuate and appear blurred. The level of blurring (contrast) is analyzed and interpreted as BP (Cutolo et al., 2017a; Ingegnoli et al., 2017). LASCA is a fast imaging technique, with a high resolution and reliability, as recently demonstrated in two studies (Lambrecht et al., 2016; Cutolo et al., 2018b).

LASCA has been applied in research studies on RP and SSc (Della Rossa et al., 2013; Ingegnoli et al., 2013, 2017) and one demonstrated that peripheral BP evaluated by both LDF and LASCA correlates to the extent of the microangiopathy (Ruaro et al., 2014).

Laser speckle contrast imaging (LCSI) is similar to LASCA and provides a five-fold increase in spatial resolution over LASCA. However, it is more time consuming (Pauling et al., 2015).

Thermal imaging (TI), an indirect method, makes use of a thermal camera to image the skin temperature to show the underlying blood flow (Clark et al., 2003; Murray et al., 2009; Pauling et al., 2012a,b; Wilkinson et al., 2018). TI evaluated RP in several studies and the response to lower temperatures (cold) was able to differentiate between PRP and SRP to SSc (Murray et al., 2009). However, it has a poor sensitivity in detecting BP variations and has a low spatial resolution (Murray et al., 2009).

Non-invasive assessment of the morphological and functional peripheral circulation may supplement the physical examination and provide a quick, accurately diagnosis, ultimately guiding the correct treatment for both PRP and SRP (Filaci et al., 1999, 2001; Faggioli et al., 2006; Pyrpasopoulou and Aslanidis, 2007; Aschwanden et al., 2008; Caramaschi et al., 2009; Miniati et al., 2009; Shah et al., 2011; Guiducci et al., 2012; Roustit et al., 2012; Cutolo et al., 2013, 2017b; Herrick, 2013, 2017; Cutolo and Sulli, 2015; Gladue et al., 2016; Smith et al., 2016b; Trombetta et al., 2016; Burmester et al., 2017; Kowal-Bielecka et al., 2017; Ruaro et al., 2017a; Rotondo et al., 2018).

Most PRP patients have no serious symptoms and respond well to conservative non-medical treatment like keeping warm and avoiding drugs with vasoconstrictive effects. Whilst other cases require pharmacological treatment like calcium channel blockers as first-line therapy (Herrick, 2013). Although various treatment options are available for the management of SSc-related SRP, these approaches at most reduce the severity of the symptoms but do not resolve the clinical situation (Herrick, 2013; Cutolo and Sulli, 2015; Gladue et al., 2016; Herrick, 2017; Kowal-Bielecka et al., 2017).

The revised European League Against Rheumatism (EULAR) recommendations for RP in SSc patients (SSc-RP) treatment state that **“*calcium channel blockers should be used as first-line therapy and PDE-5 inhibitors in patients with SSc with severe RP and/or those who do not satisfactorily respond to calcium channel blockers”*** (Kowal-Bielecka et al., 2017). The experts recommended that***“intravenous prostanoids are considered when oral therapies (including calcium channel blockers and PDE-5 inhibitors) have failed”*** and they also recognize that***“fluoxetine is a useful alternative for treatment of SSc-RP, in particular in patients with SSc who cannot tolerate or do not respond to vasodilators*”** (Kowal-Bielecka et al., 2017).

As aforementioned, the current therapies for RP are often ineffective. Therefore, the biggest challenge is identifying a drug able to halt RP progression or better still, to prevent the microvascular anomalies which involve tissue hypoperfusion and hypoxia.

That is why an NVC-based assessment of microvascular structure and an evaluation of functional impairment by laser tools and thermography may be useful to assess the efficacy of pharmacological therapies during the treatment of RP patients.

Interestingly, some studies used NVC to detect the microvascular changes as possible markers of response to immunosuppressive/anti-fibrosing treatment and vasoactive drugs (Filaci et al., 1999, 2001; Faggioli et al., 2006; Pyrpasopoulou and Aslanidis, 2007; Caramaschi et al., 2009; Miniati et al., 2009; Shah et al., 2011; Guiducci et al., 2012; Cutolo et al., 2013; Smith et al., 2016b; Trombetta et al., 2016; Ruaro et al., 2017a). Early studies on the effect of Cyclosporin have shown a moderate improvement in clinical symptoms and SSc nailfold microangiopathy, after a 12 month treatment cycle (Filaci et al., 2001; Caramaschi et al., 2009).

Similarly, Cyclophosphamide administration was reported to be significantly associated with an improvement in microvascular damage and a regression of the capillaroscopic pattern severity (Caramaschi et al., 2009).

A recent study showed no progression (therefore a positive disease modifying effect) of the microvascular damage (mainly no further capillary loss) during the 12-month follow-up in patients with early SSc and diffuse skin involvement treated with Rituximab (Smith et al., 2016b).

Recent studies have reported that the use of autologous haemopoietic stem cell transplantation in patients with severe diffuse SSc improved microangiopathy and the NVC pattern changed from “Late” to “Active” (Miniati et al., 2009). Three studies reported an improvement in nailfold microvascularization after iloprost treatment (Faggioli et al., 2006; Pyrpasopoulou and Aslanidis, 2007; Shah et al., 2011; Rotondo et al., 2018). Various studies used NVC with laser techniques to access the drug response in SSc patients treated with a combination of intravenous prostanoids and endothelin-1 receptor blockers, reporting a significant capillary loss reduction (Guiducci et al., 2012; Cutolo et al., 2013, 2014, 2016; Trombetta et al., 2016; Ruaro et al., 2017a).

The objectives of this study were:

(i) to provide a short review in the introduction on morphological (NVC) and functional techniques (laser tools and thermography) that allow for a correct early diagnosis and treatment of primary and PRP;
(ii) to present a pilot study that compares BP measured by LASCA in different skin areas of the hands and face in patients with PRP, SRP to SSc and healthy subjects (CNT).

## Patients and Methods of the Pilot Study

### Study Population

A total of 31 PRP patients were enrolled after having obtained their written informed consent for the use of imaging and the demographic data as educational material and for publications.

All the PRP patients fulfilled the LeRoy criteria (LeRoy and Medsger, 2001) as did 68 SSc patients, who met the ACR/EULAR 2013 criteria for SSc (van den Hoogen et al., 2013) during routine clinical assessment in our Rheumatology Department, from October, 2016 to Mach, 2017. The study was carried out according to the ethical standard of Good Clinical Practice. A complete medical history was collected and all participants had a clinical examination (Table 1).

**Table 1:** Clinical findings in patients with primary Raynaud’s phenomenon (PRP), systemic sclerosis (SSc) and healthy subjects (CNT).

	Median (IQR)	Median (IQR)	Median (IQR)	Median (IQR)	Median (IQR)	Median (IQR)	Median (IQR)	Median (IQR)

	CNT # 70	PRP # 31	SSc # 68	Early # 22	Active # 23	Late # 23	lcSSc # 54	dcSSc # 14
Age (years)	59 (22)	58 (24)	61 (18)	59 (20)	60 (14)	62 (13)	60 (17)	61 (14)
Gender (M/F)	4/66	1/30	3/65	1/21	1/22	1/22	3/51	1/13
Smokinghabit	3/67	2/29	3/65	2/20	1/22	0/23	2/52	1/13
RPduration (years)	NA	2 (1)	10 (8)	7 (6)	8 (7)	14 (12)	12 (8)	9 (8)
SScduration (years)	NA	NA	7 (6)	2 (2)	4 (4)	7 (7)	6 (6)	8 (6)


*The SSc study group was also classified according to the microangiopathy patterns and skin involvement (RP = Raynaud’s phenomenon; mRSS = modified Rodnan skin score; Early, Active, Late = patterns of microangiopathy at nailfold videocapillaroscopy; lcSSc = limited cutaneous SSc; dcSSc = diffuse cutaneous SSc)*.

The inclusion criteria were a diagnosis of PRP or SRP to SSc, and all patients had been on a stable drug regimen for at least 2 months prior study entry.

The exclusion criterion was being on a drug regimen that could potentially influence blood flow.

If the patients were being treated with prostanoids and endothelin-1 receptor antagonists, they were temporarily withdrawn 1 month before instrumental assessment.

All SSc patients were taking aspirin (average dosage 100 mg/day) at the time of the study. Other concomitant treatment included: proton pump inhibitors (used by #52 patients), antihypertensive drugs i.e., angiotensin-converting enzyme (ACE) inhibitors (#9 patients), cyclosporine (average dosage 150 mg/day: #12 patients), methotrexate (average dosage 7.5 mg/week: #12 patients). The PRP therapy treatment was: proton pump inhibitors (used by #8 patients), antihypertensive drugs i.e., ACE inhibitors (#3 patients).

Both LASCA and NVC were performed on the same day in all PRP and SSc patients.

Laser speckle contrast analysis was also performed in the 70 healthy subjects (CNT) matched with the RP patients for age and gender (see Table 1 for demographic data).

### Laser Speckle Contrast Analysis (LASCA)

**FIGURE 1:**
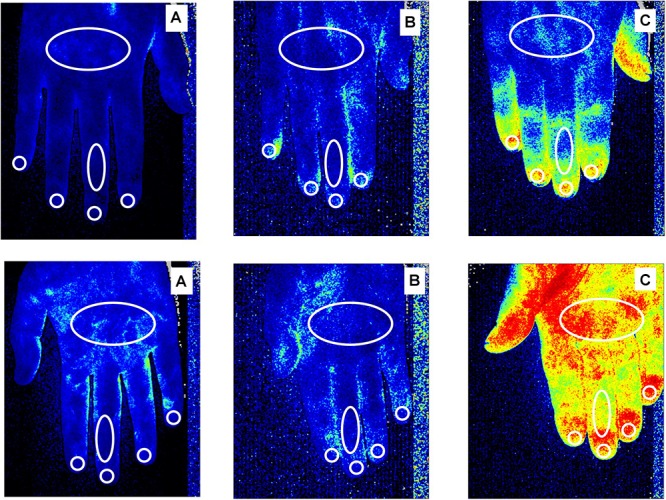
Laser Speckle Contrast Analysis (LASCA) images of secondary Raynaud’s phenomenon (RP) to systemic sclerosis, in a patient with a “Late” pattern of scleroderma microangiopathy **(A)**, primary RP **(B)** and a healthy subject **(C)**, showing the regions of interest (ROI - white circles) created at the level of dorsum and palm of the hand, dorsal and palmar aspect of the 3rd finger, periungual areas and fingertips to evaluate blood perfusion. Color code: blue corresponds to a low BP, yellow an intermediate BP and red a higher BP. Noteworthy is the fact that subjects with a late pattern have a prevalence of blue, indicating a low perfusion level.

Skin BP was analyzed by the LASCA technique (Pericam PSI, Perimed, Milan, Italy) at the level of dorsal and palmar aspect of hands and the whole face, in both SSc patients and healthy subjects as previously described (Ruaro et al., 2014, 2016; Sulli et al., 2014). Different regions of interest (ROIs) were created, as previously reported, i.e., at the level of fingertips, periungual areas, dorsal and palmar aspect of the 3rd finger bilaterally, the dorsum and palm of both hands and face (forehead, tip of nose, zygomas and perioral region) (see Figure 1 for ROI areas) (Sulli et al., 2014; Ruaro et al., 2016, 2018b).

The average BP from either fingertips or periungual areas was calculated by summing the perfusion values of eight fingers together and then dividing the final value by the number of fingers.

The average BP from the two palmar and dorsal areas of the fingers, palm and dorsum of the hands and zygoma was calculated by summing the perfusion values of the two sides (right and left) and then dividing the final value by two. The BP was quantified as perfusion units (PU; Sulli et al., 2014; Ruaro et al., 2016). The same operator (BR) performed the examination in all PRP, SRP-SSc patients and CNT.

### Nailfold Videocapillaroscopy (NVC)

All patients were assessed by nailfold videocapillaroscopy (NVC), (equipped with a 200× contact lens, connected to image analysis software – Videocap, DS MediGroup, Milan, Italy) so as to distinguish PRP from SRP and to determine the correct nailfold microangiopathy pattern (“Early,” “Active,” or “Late” pattern, according to the Cutolo’s criteria) in the SSc patients (Sulli et al., 2008; Smith et al., 2010, 2013; Table 1). The same operator (CP) performed the examination in all PRP and SRP-SSc patients and CNT.

### Statistical Analysis

The statistical analysis was carried out by parametric procedures and confirmed by non-parametric tests. The Mann-Whitney *U* test was performed to compare unpaired groups of variables, along with the Kruskal-Wallis test to compare continuous variables with nominal variables that had more than two levels. Any *p*-values below 0.05 were considered statistically significant. The results are given as, median and interquartile range (IQR).

## Results

Both PRP and SSc patients had statistically significant lower BP than the healthy subjects at the fingertip (*p* < 0.0001), the periungual area (*p* < 0.0001), the palmar aspect of the 3rd finger (*p* < 0.0001) and the palm areas (*p* < 0.0001). Conversely, all three groups had similar BP values in the other areas of the hand (dorsal aspect of the 3rd finger and dorsum of hand) and face (forehead, tip of nose, zygomas and perioral region). Moreover, BP was statistically significantly lower in PRP than in SSc patients with the “Early” pattern of microangiopathy at fingertip (*p* = 0.04), periungual (*p* < 0.05), palmar aspect of the 3rd finger (*p* = 0.0008) and the palm areas (*p* = 0.0009). No statistically significant difference was observed between PRP and the “Early” pattern of microangiopathy in the other areas evaluated.

A statistically significant progressive decrease in BP was confirmed in SSc patients with a progressive pattern of nailfold microangiopathy (“Early,” “Active,” and “Late”) at the fingertip, periungual, palmar aspect of the 3rd fingers and palm areas (*p* < 0.05). No statistically significant difference was observed between NVC patterns and BP at the level of the other areas (dorsum of hands, whole face and different areas of face) (*p* > 0.05) (Table 2).

**Table 2:** Blood perfusion (BP) in systemic sclerosis (SSc), primary Raynaud’s phenomenon (PRP) and healthy subjects (CNT).

	Median (IQR)	Median (IQR)	Median (IQR)	Median (IQR)	Median (IQR)	Median (IQR)	Statistical significance					
	CNT # 70	PRP # 31	SSc # 68	Early # 22	Active # 23	Late # 22	CNT vs. PRP	CNT vs. SSc	PRP vs. SSc	E vs. A	E vs. L	A vs L
BP fingertips	187 (72)	90 (28)	88 (25)	92 (58)	88 (19)	82 (40)	*p* < 0.0001	*p* < 0.0001	*p* = 0.6	*p* = 0.2	*p* = 0.006	*p* = 0.1
BP palmar aspect of the 3rd phalanx	134 (74)	84 (19)	81 (27)	88 (25)	80 (20)	72 (35)	*p* < 0.0001	*p* < 0.0001	*p* = 0.4	*p* = 0.06	*p* = 0.007	*p* = 0.2
BP palm of hands	114 (27)	81 (22)	79 (31)	85 (22)	83 (31)	68 (39)	*p* < 0.0001	*p* < 0.0001	*p* = 0.05	*p* = 0.4	*p* = 0.01	*p* = 0.04
BP periungual areas	143 (51)	78 (28)	76 (38)	82 (34)	76 (47)	68 (42)	*p* < 0.0001	*p* < 0.0001	*p* = 0.7	*p* = 0.1	*p* = 0.02	*p* = 0.3
BP dorsal aspect of the 3rd phalanx	55 (28)	59 (16)	58 (24)	61 (19)	59 (25)	57 (24)	*p* = 0.5	*p* = 0.09	*p* = 0.8	*p* = 0.2	*p* = 0.3	*p* = 0.6
BP dorsum of hands	51 (27)	50 (13)	52 (18)	56 (22)	50 (19)	49 (16)	*p* = 0.4	*p* = 0.07	*p* = 0.9	*p* = 0.1	*p* = 0.1	*p* = 0.8
BP forehead	109 (44)	113 (32)	110 (33)	112 (21)	110 (29)	111 (31)	*p* = 0.1	*p* = 0.09	*p* = 0.3	*p* = 0.2	*p* = 0.3	*p* = 0.8
BP tip of nose	129 (45)	139 (42)	130 (42)	132 (42)	129 (36)	130 (56)	*p* = 0.2	*p* = 0.09	*p* = 0.3	*p* = 0.5	*p* = 0.09	*p* = 0.3
BP zygoma	127 (48)	155 (45)	145 (58)	150 (45)	145 (55)	143 (83)	*p* = 0.4	*p* = 0.1	*p* = 0.2	*p* = 0.3	*p* = 0.2	*p* = 0.1
BP perioral region	144 (48)	141 (39)	135 (46)	134 (45)	136 (46)	134 (56)	*p* = 0.1	*p* = 0.1	*p* = 0.3	*p* = 0.2	*p* = 0.1	*p* = 0.3
BP whole face	135 (34)	146 (28)	136 (42)	140 (32)	131 (36)	130 (68)	*p* = 0.2	*p* = 0.3	*p* = 0.1	*p* = 0.2	*p* = 0.1	*p* = 0.2


*SSc patients with different capillaroscopic patterns of nailfold microangiopathy (Early, Active, Late), evaluated by LASCA in different areas of the hands (fingertips, periungual areas, dorsal and palmar aspect of the 3rd phalanx bilaterally, dorsum and palms of both hands) and face (forehead, tip of nose, zygomas and perioral region). Statistical significance: Mann-Witney *U*-test (# = numbers, E = Early, A = Active, L = Late). The statistical significance columns show the blood perfusion difference between the CNT (healthy subjects) and the PRP (primary Raynaud’s phenomenon) and the systemic sclerosis (SSc) patients. The three capillaroscopic patterns of nailfold microangiopathy (Early, Active, Late), evaluated by LASCA, are also documented*.

If the three nailfold microangiopathy patterns (“Early,” “Active,” and “Late”) are evaluated separately, there is a statistically significant difference only between the “Early” and “Late” group, at the level of the fingertip, periungual, palmar aspect of the 3rd fingers and palm areas (*p* < 0.05). No statistically significant difference was observed in the other areas.

There were very few smokers in our study and there was no statistically significant difference in the smoking habit between the groups.

## Discussion

Our pilot study shows that the hand BP, evaluated by LASCA, was lower in PRP than in SSc patients with an “Early” NVC microangiopathy pattern.

The results of this study also confirm that SSc patients had a significant lower median BP than healthy subjects and the progressive decrease of BP in SSc patients with different: “Early,” “Active,” or “Late” NVC pattern of microangiopathy at the level of hand.

Indeed, some authors have reported different perfusion values in PRP and SRP to SSc patients, but the perfusion was evaluated either after, or during, different forms of stress, such as the cold or occlusion test, in contrast with our study where the perfusion was evaluated at basal condition (Pauling et al., 2012a, 2015).

We would like to attest that all the PRP patients had a functional disorder/dysfunction in microvascular circulation and our data emphasize the importance of the perfusion reduction, even in a functional phenomenon such as in PRP patients.

Moreover, our data are in agreement with those of other studies that report NVC as being the best method to evaluate microcirculation morphological and permanent damage and to make a differential diagnosis between PRP and SRP (Murray et al., 2009; Ingegnoli et al., 2017; Herrick and Murray, 2018).

As previously reported our data confirm that patients with the “Late” SSc microangiopathy pattern had a lower blood flow than those with the “Active” or “Early” SSc patterns at NVC (Ruaro et al., 2014, 2018b). In our precedent article we also reported that when BP was assessed by the LASCA technique significantly lower values were observed in the SSc patients than in the healthy subjects at the level of the fingertips, periungual areas and palm of the hands, with a statistically significant negative correlation between the extent of the nailfold microangiopathy and the BP values at the level of the same skin areas in SSc patients (Ruaro et al., 2014, 2018b).

The increased interest in microcirculation has led to a rapid development of new assessment methods. However, these techniques lack the support of validation studies as to their application in clinical practice. Nevertheless, microvascular structure evaluation by NVC combined with functional investigation by laser techniques or TI, not only helps in the distinction between primary and SRP, but is also able to evaluate therapy response and disease progression (Filaci et al., 2001; Caramaschi et al., 2009; Guiducci et al., 2012; Cutolo et al., 2013, 2014, 2016; Smith et al., 2013, 2016b; Trombetta et al., 2016; Ruaro et al., 2017a, 2018a, Pizzorni et al., 2017a; Soulaidopoulos et al., 2017; Markusse et al., 2017).

In particular, the assessment of the number of capillary changes seems the best validated NVC parameter and is today evaluable with automated systems (Cutolo et al., 2018a).

In summary we are of the opinion that morphological evaluation by NVC is the best method for the early detection and quantification of microvascular abnormalities that characterize SRP. We also believe that clinicians should not underestimate RP which should have a scheduled follow-up as it might well be a precocious *cloaked clinical sign* of abnormal microcirculation and a risk factor for the development of a CTD, especially SSc.

Last but not least, the main message of this work is that while today there is no curative treatment all RP patients, because it is a very heterogeneous phenomenon, still there are many treatment options to improve quality of life of these patients. The early detection of disease and immediate intervention appears to make a difference, such as well-designed clinical trials and collaboration with networks, such as the European Reference Network on Rare and Complex Connective Tissue and Musculoskeletal Diseases Project and specialized centers carrying the research in this field with the aim of defining ideal diagnostic and therapeutic options (Smith et al., 2018).

## Ethics Statement

This study has been performed in accordance with the ethical standards laid down in the 1964 Declaration of Helsinki and its later amendments. Ethics approval was obtained from the local Ethical Board and all patients gave written informed consent to enter the study.

## Author Contributions

All authors listed have made a substantial, direct and intellectual contribution to the work, and approved it for publication.

## Conflict of Interest Statement

The authors declare that the research was conducted in the absence of any commercial or financial relationships that could be construed as a potential conflict of interest.
